# Optimizing Friction Stir Spot Welding Parameters for Enhanced Mechanical, Thermal, and Microstructural Properties of PA6

**DOI:** 10.3390/polym17182508

**Published:** 2025-09-17

**Authors:** Ayşe Danış Bekdemir, İdris Karagöz

**Affiliations:** 1 Department of Polymer Materials Engineering, Institute of Graduate Studies, Yalova University, Yalova 77200, Türkiye; 2 Department of Polymer Materials Engineering, Faculty of Engineering, Yalova University, Yalova 77200, Türkiye

**Keywords:** friction stir spot welding, PA6, polymer joining, plastic welding, welding parameters

## Abstract

The objective of this study is to systematically investigate the effects of friction stir spot welding (FSSW) parameters—rotational speed, dwell time, and pin diameter—on the mechanical, thermal, and microstructural properties of PA6. PA6 plates (5 mm thick, 30 mm wide, 150 mm long) were welded using an Optimum BF20L milling machine, examining key parameters: rotational speed (762, 1146, 1560 rpm), pin diameter (M10, M12), and dwell time (15 s, 60 s). A full factorial design was employed to analyze their effects. Rotational speed emerged as the most significant factor influencing tensile strength, with an optimal speed of 1146 rpm yielding 72.4 MPa. Dwell time also played a major role, improving flexural strength by 56.5% as it increased from 15 to 60 s (40.6 MPa to 63.6 MPa). Although pin diameter had limited influence on tensile performance, larger pins (M12) promoted higher crystallinity (up to 33.37%) and better thermal distribution. The degree of crystallinity and crystalline lamella thickness (λ) varied, indicating that thermal and structural properties can be tailored through parameter optimization. These findings highlight the potential of FSSW to enhance PA6’s performance characteristics, making it a viable joining method for high-performance applications in the automotive, aerospace, and electronics industries. Further research is encouraged to deepen the understanding of the relationship between welding parameters and microstructural evolution, particularly in relation to crystallization behavior.

## 1. Introduction

Polyamide 6 (PA6) stands out among engineering plastics due to its high tensile and impact strength, its ability to maintain structural stability across a wide temperature range, and its strong resistance to oils, greases, aliphatic hydrocarbons, and many solvents [1]. These properties make PA6 a popular choice in industries such as automotive, electrical and electronics, packaging, and textiles. In the automotive industry, for instance, PA6 is used in engine parts, fuel systems, gears, and pipelines. In electrical and electronics applications, it is commonly found in connectors, plugs, and terminals. Its low oxygen permeability makes it ideal for packaging, and in textiles, PA6 is valued for its flexibility, durability, and abrasion resistance in fiber form. Additionally, PA6’s superior mechanical properties make it a preferred material in the production of composite structures [2].

Despite these advantages, the manufacturing process for PA6 poses challenges, particularly when producing complex parts. While methods like injection molding allow for the production of many PA6 components, they are not always sufficient for the creation of more intricate designs. Often, semi-finished forms of PA6, such as sheets, profiles, or pipes, need to be joined to form more complex parts [3]. For this reason, the joining of plastics, especially PA6, is of critical importance in industries such as automotive, medical, aerospace, and packaging, where structural integrity and precision are paramount [4]. Welding is one of the most common methods for joining plastic components. PA6 components are typically joined using methods such as hot plate welding, ultrasonic welding, and friction stir welding, all of which produce strong, reliable joints without compromising the material’s properties [5]. However, these traditional welding methods can sometimes cause undesirable effects, such as deformation and structural degradation, as the material melts during the process. In addition to thermal and mechanical joining methods, chemical techniques such as adhesive bonding and solvent welding are widely used for joining PA6 and other thermoplastics. These approaches can provide strong joints under moderate conditions, but they often require surface pretreatments and exhibit limitations in thermal stability, environmental durability, and long-term performance [3]. In this context, FSSW emerges as a solvent-free and environmentally friendly alternative that avoids the use of additional chemicals while offering stable and reliable weld quality [5]. In recent years, friction stir spot welding (FSSW) has emerged as an innovative alternative, having been successfully adapted from the metal industry for use with plastics. This method not only produces high-quality weld zones but also offers additional advantages like energy efficiency, low thermal impact, and an environmentally friendly process.

Given the increasing use of PA6 in high-performance applications, the development of effective joining methods is essential. As the adoption of FSSW for plastics has grown, research on its applicability to PA6 has become a critical area of focus [6,7,8,9,10,11]. Understanding the parameters that influence weld quality is vital to optimizing this process. In the joining of PA6 using FSSW, numerous factors affect weld strength and quality. These include tool rotation speed [10,11,12], preheating (dwell time), stirring and cooling time [10,11,12,13,14,15], plunge depth [10], tool shoulder and pin diameter [16,17,18], and tool pin geometry [9,12,14]. Additionally, the effects of filler materials on weld strength have been studied [12,18]. However, despite these investigations, researchers agree that the current understanding of FSSW for thermoplastics, particularly PA6, is still insufficient and that more studies are required to advance the technique and achieve widespread industrial use [4,6,7,8].

Among the parameters influencing FSSW performance, the plunge rate has been identified as particularly crucial. Yan et al. proposed that determining the optimal plunge rate is essential for achieving high weld strength. Low plunge rates negatively affect weld strength by reducing the material’s interaction time and the size of the welded region, which lowers the load-bearing capacity. Conversely, excessively high plunge rates can disrupt the balance between material stirring and heating, also leading to weaker welds [11]. The plunge depth, another important factor, controls the penetration and geometry of the weld. As the plunge depth increases, the bonding area between the materials expands. However, both too shallow and too deep of a plunge depth can reduce tensile strength, indicating that an optimal range must be found for strong, well-formed welds [19]. Additionally, during the FSSW process, issues like bubble formation in the weld region may arise due to the polymer’s moisture content and air trapped during the plunge phase. The axial force applied during plunging compresses the material, reducing bubble formation and improving the weld’s mechanical performance by increasing the load-bearing capacity [20].

Research suggests that preheating time has a minimal impact on overall weld quality. While preheating primarily affects the upper surface region beneath the welding tool, its effect on the lower layers is limited due to the low thermal conductivity of polymers [21,22,23]. More significant is the dwell time, which influences weld strength by determining the depth and size of the weld. Longer dwell times can enhance weld strength by increasing weld depth, but if extended too long, they may cause adverse effects such as material expulsion, void formation, and reduced molecular weight due to mechanical degradation of polymer chains [11]. Therefore, balancing the dwell time to achieve the desired weld strength is essential. At the end of the plunging and stirring process, the heat generated in the weld zone softens the polymers, forming an elastic structure. This structure requires a specific cooling time due to the low thermal diffusivity of polymers [8]. The cooling time depends on factors such as the maximum temperature reached during the welding process and the thermal properties of the material, including the glass transition temperature (Tg). As the material cools, the tool should be withdrawn at an appropriate moment to avoid weakening the weld zone.

The size of the shoulder and pin also plays a significant role in determining weld quality. Larger tool shoulder diameters increase tensile strength by enlarging the stirred region, while larger tool pin diameters can reduce weld strength due to unfavorable changes in the stirred area’s characteristics [8]. Additionally, the geometry of the tool pin is a critical factor. Studies have shown that a threaded cylindrical pin outperforms a plain cylindrical pin in producing stronger welds, following the same principles seen in FSSW of metals like aluminum [23].

In summary, the most influential parameters in the FSSW of PA6 are the dwell time and the rotational tool. However, the literature presents conflicting results on the effect of tool rotational speed, with some studies suggesting minimal influence or even a reduction in weld strength at high speeds, while others indicate that an optimal rotational speed enhances weld quality [11,24]. Lambiase et al. emphasized the non-linear relationship between tool rotational speed and weld quality, attributing this to the temperature sensitivity of polymers. Factors such as material thickness, tool dimensions, and the thermal properties of the polymer all play a role in determining the ideal welding conditions [8].

The use of advanced polymers like PA6 in high-performance applications is rapidly increasing due to their excellent mechanical properties, resistance to a wide range of chemicals, and thermal stability. In industries such as automotive, aerospace, and electronics, reliable and efficient joining methods are essential to ensure structural integrity. While PA6 is often processed via injection molding, joining techniques are crucial for more complex assemblies. However, traditional welding methods can lead to degradation and structural changes that affect the material’s performance. Friction stir spot welding (FSSW) has emerged as a promising alternative, especially for thermoplastics like PA6. This technique produces high-quality joints with lower thermal impact than conventional methods. Despite its potential, comprehensive research on the influence of FSSW parameters on PA6 welds remains limited, hindering its wider adoption. Studies suggest that factors like tool rotational speed, dwell time, and pin geometry significantly impact weld quality. Yet, optimal conditions for PA6 welding are not well established, leading to conflicting results in the literature. Although previous studies have investigated the feasibility of FSSW on PA6, there is still a lack of systematic analyses that simultaneously evaluate the effects of rotational speed, dwell time, and pin diameter on the mechanical, thermal, and microstructural properties of the joints. This study addresses this gap by employing a full factorial design to quantify both the main and interaction effects of these parameters, thereby providing new insights into the optimization of FSSW for PA6.

In this study, PA6 materials will be joined using the FSSW method, with varying tool rotation speeds, dwell times, and pin diameters. Unlike conventional FSSW tools that typically employ small pin diameters and large shoulder diameters, this study introduces a novel tool design featuring larger pin diameters (M10 and M12) and shoulder diameters twice the size of the pin diameter. The mechanical, thermal, and microstructural properties of the resulting welds will be thoroughly examined to assess the impact of this unique tool design on weld performance. This investigation aims to provide valuable insights for optimizing the FSSW process for PA6, particularly in exploring the potential of such tool configurations, thereby enhancing its application in industries where high-performance, durable joints are crucial.

## 2. Materials and Methods

### 2.1. Materials

In the FSSW process, PA6 plates with a thickness of 5 mm, a width of 30 mm, and a length of 150 mm were used (density: 1.13 g/cm^3^, water absorption: 0.9%, hardness: 82 Shore D, coefficient of friction: 0.4, melting temperature: 220 °C, notched impact strength: 6 kJ/m^2^). The FSSW applications were performed on an Optimum brand BF20L model desktop milling machine (Optimum Maschinen GmbH, Hallstadt, Germany). The welding process utilized a traditional aluminum-style welding tool, which was machined from 42CrMo4 (1.7225) material (shoulder diameter: Ø 20 mm, pin diameter: M10 and M12, pin length: 8 mm). The welding parameters were determined using ANOVA analysis in the Minitab 17 program, as shown in Table 1.

### 2.2. Full Factorial Design Analysis and Joining PA6 Sheets Using FSSW

A full factorial design of experiments, summarized in Table 2, was developed to assess the performance of welded joints in the friction stir spot welding (FSSW) of PA6 sheets and to evaluate the main effects and interactions of tool rotational speed, dwell time, and pin diameter on their strength. The design consists of three factors: rotational speed (762 rpm, 1146 rpm, and 1560 rpm), dwell time (15 s and 60 s), and pin diameter (10 mm and 12 mm), enabling a comprehensive analysis of their effects and interactions. Three levels were selected for rotational speed to capture its potentially non-linear influence on joint performance, as this parameter significantly affects heat generation and material flow during welding. In contrast, two levels were deemed sufficient for dwell time and pin diameter, as prior studies indicate their effects tend to follow linear or near-linear trends within the selected ranges, thus balancing experimental thoroughness with resource efficiency. For PA6, preliminary trials further confirmed that dwell times shorter than 15 s led to insufficient stirring, whereas those longer than 60 s caused excessive flash and thermomechanical degradation. Similarly, the selected pin diameters (M10 and M12) represent a practical range for 5 mm sheets; smaller pins resulted in weak mixing, while larger pins risked overheating and thinning. Therefore, two levels were sufficient to capture their influence without entering impractical processing windows.

**Figure 1:**
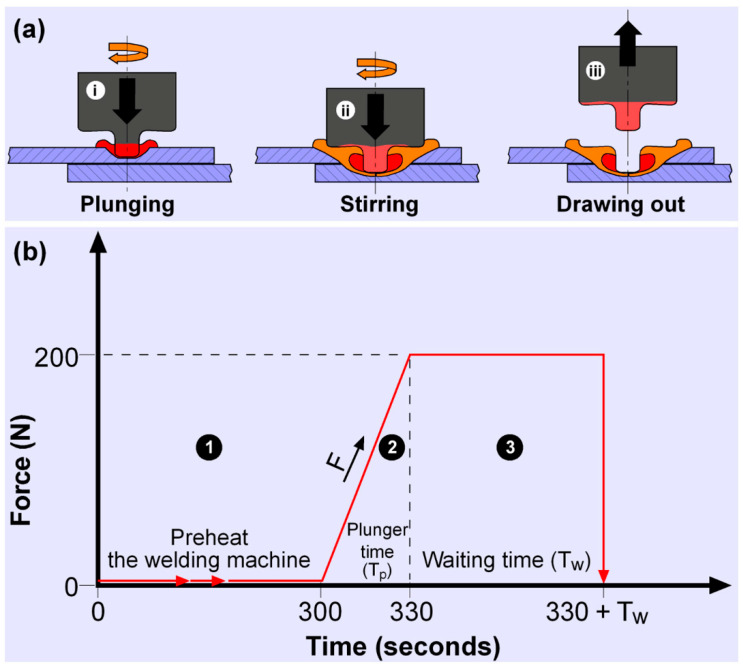
Schematic representation of the FSSW process: (**a**) basic principle and (**b**) sequential steps of the method.

**Figure 2:**
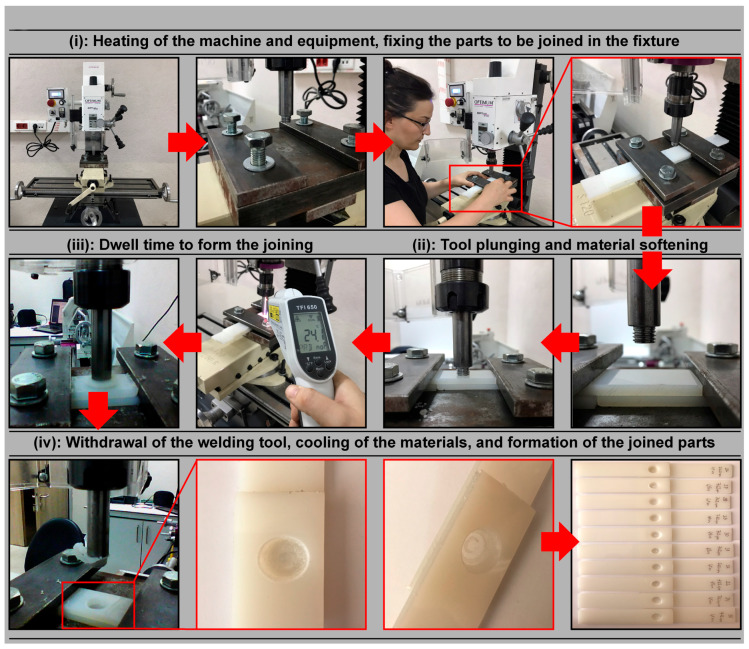
Schematic representation of the FSSW application stages, including (**i**) plate stacking, (**ii**) plunging, (**iii**) dwelling, and (**iv**) tool retraction.

FSSW, as shown in Figure 1a, is a solid-state welding method in which a rotating tool contacts the surface of the workpiece to generate frictional heat, plunges into the material to join it through mechanical stirring, and finally completes the process by retracting upward. The joining processes using FSSW were carried out according to the process steps illustrated in Figure 1, with a schematic representation of the FSSW application in this study shown in Figure 2.

Initially, the plates prepared for joining were stacked, as shown in Figure 2i, with the joining areas overlapped and secured to a specially designed fixture using bolts. At the start of the welding process, the milling machine was operated for 5 min to heat the tool and equipment (Figure 1, Stage 1). A rotating tool (a conventional aluminum-style welding tool) was plunged into the material surface with a constant force of 200 N, controlled by the milling machine’s calibrated axial force control system and maintained within ±2 N throughout the process, and a speed of 0.267 mm/s (Figure 1, Stage 2), as depicted in Figure 2ii. During this phase, the tool’s shoulder generated heat through friction on the material surface, while the pin penetrated and stirred the material, causing it to soften and become plastic. Once the desired plunge depth was achieved, the tool continued to rotate steadily during the dwell time in the weld zone (Figure 1, Stage 3), as shown in Figure 2iii. During this time, the pin stirred the material, creating a strong mechanical bond at the contact surfaces of the two plates. At the end of the dwell time, the tool was withdrawn, allowing the welded area to cool, as illustrated in Figure 2iv. In addition, thermal monitoring was conducted using an infrared thermal camera (FLIR E8, FLIR Systems, Wilsonville, Oregon, USA) to verify the heat distribution and peak temperatures in the weld zone during plunging and dwelling, ensuring that the thermal input remained within a safe range for PA6. The use of this thermal camera is justified as it allowed us to non-invasively track the temperature field, detect possible overheating, and correlate thermal profiles with mechanical and microstructural results.

### 2.3. Characterization

The lab shear test (ISO 527-1) [25] and the three-point bending test (ISO 178) [26] were both conducted at room temperature using a Zwick Z020 model testing machine (ZwickRoell GmbH&Co. KG, Ulm, Germany). A crosshead speed of 50 mm/min was applied for the lab shear test, while a bending rate of 1.7 mm/min was used for the three-point bending test. For each welding condition, five specimens were tested, and the reported values represent the mean with corresponding standard deviations to ensure repeatability.

In order to investigate the thermal transitions of the welded specimens, differential scanning calorimetry (DSC) analysis was conducted using a Setaram DSC131 Evo instrument (Setaram Instrumentation, Cailuire, France), following the ASTM D3417 standard [27] protocol. The measurements were carried out under a nitrogen atmosphere, with a constant heating rate of 10 °C/min. Based on the obtained DSC thermograms, the degree of crystallinity (Xc) was determined using Equation (1), while the thickness of the crystalline lamellae (λ) was calculated through the Gibbs–Thomson equation, given in Equation (2). For PA6, a reference melting enthalpy (ΔH°m) of 230 J/g corresponding to a fully crystalline structure was adopted, along with an equilibrium melting point (Tm°) of 146 °C and a crystal surface free energy (δe) value of 11.4 mJ/m^2^ [28,29,30,31,32]. In these expressions, ΔHm represents the experimental melting enthalpy, Tm indicates the observed melting peak temperature, and ϕi denotes the mass fraction of PA6 in the analyzed sample.

(1) $$X_{c}\%=\left(\;\frac{\Delta Hm}{\Delta H{}^{\circ}m\times \phi i}\right)\times 100$$

(2) $$T_{m}=Tm{}^{\circ}\left(1-\frac{2\delta e}{\lambda \Delta H_{m}}\right)$$

## 3. Results

### 3.1. Lab Shear Test Results for Sheets Joined by FSSW

**Figure 3:**
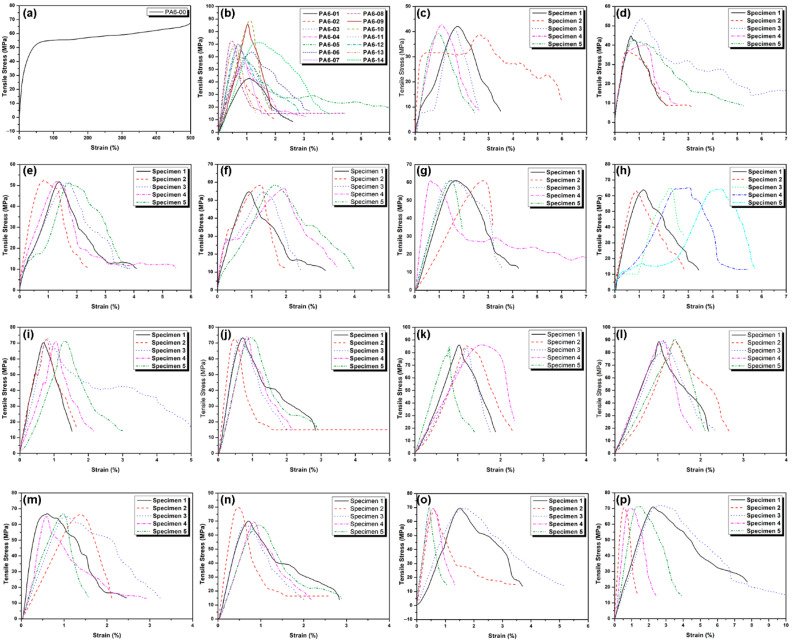
Graphical comparison of lab shear test results: (**a**) PA6-00 (pure PA6), (**b**) all samples joined by FSSW, (**c**) PA6-01, (**d**) PA6-02, (**e**) PA6-03, (**f**) PA6-04, (**g**) PA6-05, (**h**) PA6-06, (**i**) PA6-07, (**j**) PA6-08, (**k**) PA6-09, (**l**) PA6-10, (**m**) PA6-11, (**n**) PA6-12, (**o**) PA6-13, (**p**) PA6-14.

The graphs of the lab shear test results are presented in Figure 3a–p. The lab shear properties of PA6 sheets subjected to the FSSW process, including elastic modulus, shear tensile strength, fracture strength, and elongation at break, exhibit significant differences, as summarized in Table 3. These differences highlight the effects of processing parameters (dwell time, rotational speed, and pin diameter) on the material’s mechanical performance [33,34].

Regarding elastic modulus, the PA6-00 (reference) sample displays the lowest value at 3200 MPa (Figure 3a), while the PA6-13 sample reaches the highest value at 10,400 MPa (Figure 3o). As rotational speed increases, a general rise in elastic modulus values is observed. The highest elastic modulus in PA6-13, achieved with a high rotational speed (1560 rpm), demonstrates that the material becomes stiffer and more resistant to deformation. In other samples, particularly when pin diameter and dwell time vary, elastic modulus values range from 7000 MPa to 10,000 MPa (Figure 3c–n), indicating that processing parameters significantly influence the material’s elasticity.

In terms of tensile strength, the PA6-00 sample exhibits the highest strength at 73.4 MPa (Figure 3a), while samples such as PA6-01 and PA6-02 show a reduction to around 40 MPa (Figure 3c,d). This decrease is particularly linked to low pin diameter and short dwell times, which fail to sufficiently homogenize the material structure during the FSSW process, resulting in reduced tensile strength [8]. However, in samples like PA6-09, PA6-10, and PA6-13 (Figure 3i,j,o), where rotational speed and pin diameter are increased and dwell times are longer, tensile strength remains above 70 MPa, indicating better performance. Specifically, the combination of high rotational speed and long dwell time in PA6-13 enhances its strength.

For fracture strength, PA6-00 again shows the highest value at 70.4 MPa (Figure 3a), while samples like PA6-01 and PA6-02 have significantly lower fracture strength (around 8 MPa) (Figure 3c,d). This is due to insufficient thermal effects generated by short dwell times and small pin diameters [11,15,16,17,18]. Short mixing times prevent proper material mixing, leading to reduced fracture strength. In contrast, longer dwell times and adequate rotational speed overcome this issue, resulting in better fracture strength in samples like PA6-03 and PA6-04 (Figure 3e,f).

In terms of elongation at break, PA6-00 exhibits the highest value at 540% (Figure 3a), while other samples show much lower values, such as 1.3% for PA6-01 and 0.8% for PA6-02 (Figure 3c,d). This reduction in elongation is due to the reduced flexibility of the weld zone, which is influenced by changes in pin diameter, rotational speed, and dwell time [10,18]. High rotational speeds and long dwell times can lead to a denser material structure, causing a loss of flexibility. However, in samples like PA6-09, PA6-10, and PA6-13 (Figure 3i,j,o), with shorter dwell times and suitable rotational speeds, elongation at break remains relatively higher.

**Figure 4:**
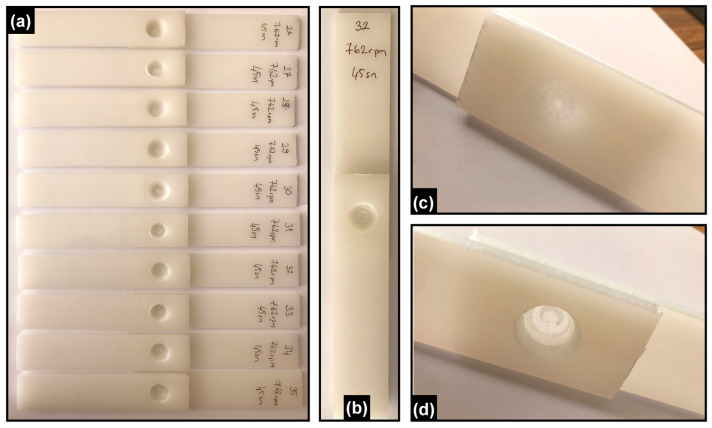
Cross-sectional and surface views of the welded joints: (**a**) overview of joints produced under selected process parameters, (**b**) a single welded joint, (**c**) the back surface, and (**d**) the front surface of the joint.

In addition, representative cross-sectional and surface views of the welded joints are presented in Figure 4. These include (a) an overview of joints produced under selected process parameters, (b) a single welded joint, (c) the back surface, and (d) the front surface of the joint. These images provide complementary evidence of the weld geometry and morphology, supporting the interpretation of the mechanical test results.

Overall, significant changes in the mechanical properties of PA6 sheets welded by the FSSW process have been observed. The PA6-00 (reference) sample performs better, with a higher elastic modulus, tensile strength, fracture strength, and elongation at break compared to other samples, while FSSW-processed samples show a marked decrease in mechanical properties. However, in samples like PA6-13 and PA6-14, although the elastic modulus, tensile strength, and fracture strength are high, elongation at break remains low, indicating these samples are stiffer but less flexible. The superior elongation of the PA6-00 reference sample can be explained by the absence of weld-induced defects and the preservation of the polymer’s inherent ductility. In welded samples, voids and weak interfacial bonding arising from insufficient material flow (short dwell times or small pin diameters) reduce strength, while densification of the weld zone at longer dwell times improves strength but causes a significant loss of ductility. In some cases, excessive heat input may even induce thermomechanical degradation of the polymer. Nevertheless, under optimized conditions—particularly at 1146 rpm, 60 s dwell time, and M12 pin diameter—the tensile and shear strengths approach those of the reference sample, showing that welding can enhance performance when material flow and heat input are properly balanced.

These findings demonstrate that the mechanical properties of PA6 sheets welded by the FSSW process are dependent on welding parameters (such as dwell time, rotational speed, and pin diameter), and these parameters have significant effects on the material’s overall performance. Specifically, as rotational speed and dwell time increase, the material’s stiffness and strength improve, but its flexibility and elongation at break decrease [10,12]. This emphasizes the importance of optimizing parameters to make FSSW-processed materials more suitable for specific applications.

**Figure 5:**
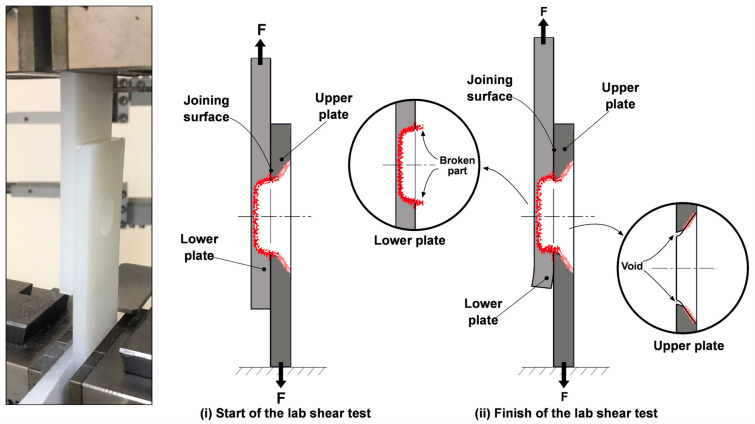
Schematic representation of lab shear test: start (**i**) and end (**ii**) stages.

In Figure 5, the start of the lab shear test (Figure 5i) and the images after the test (Figure 5ii) are shown schematically. At the start of the test, the joining surface is compressed between the upper and lower plates, with the test force (F) applied in opposite directions on both plates. At this stage, the material is in the elastic or plastic deformation region, and no breakage or voids are observed. However, by the end of the test, breakage occurs in the welding zone. The red line, representing the breakage path (broken part), marks the point where the break occurs on the joining surface (the weak point). Additionally, voids are observed on the joining surfaces of the upper plate. These voids may indicate that the material in the welding zone did not bond sufficiently homogenously with the base material, negatively affecting its mechanical performance. The weakness in the welding zone led to breakage predominantly in this area, reducing the material’s strength. Factors such as insufficient welding temperature, low pressure, or the entrapment of gases in the material could cause the formation of these voids. Overall, this phenomenon can be explained by the fact that, at this point, the plate joining surfaces overlap, causing gases generated by the heat in the welding zone to try to escape from the joining line [8,11]. This leads to void formation in the region and negatively impacts the weld strength. Such breakages and voids in the joining region indicate that the mechanical strength of the welding zone is low, and that the welding parameters (e.g., pressure, temperature, and duration) need to be properly optimized [19,21,22,23].

**Figure 6:**
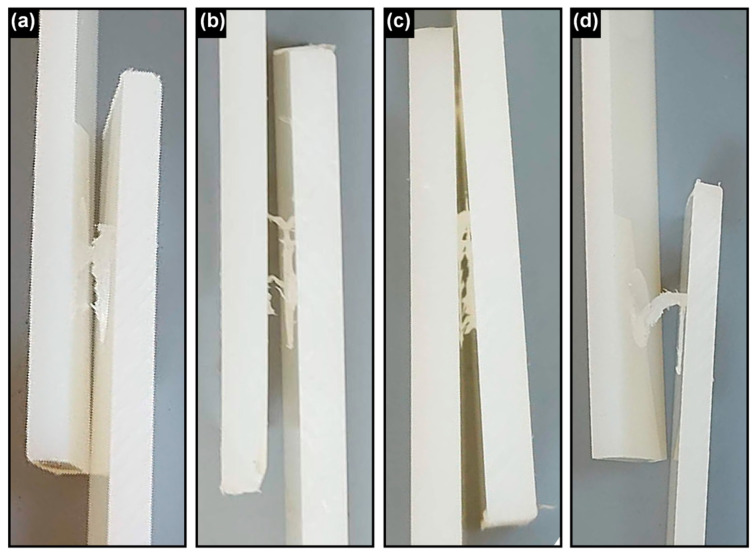
Failure paths of welded PA6 joints under different dwell times: (**a**) PA6-02, (**b**) PA6-03, (**c**) PA6-04, and (**d**) PA6-06. Cracks propagated differently depending on dwell time, ranging from interfacial separation to cohesive fracture within the stir zone.

To further illustrate the fracture mechanisms, representative failure paths of welded joints are presented in Figure 6. As seen in Figure 6a (PA6-02), cracks propagated mainly along the weakly bonded interface, indicating adhesive-type failure. In Figure 6b,c (PA6-03 and PA6-04), longer dwell times promoted better mixing, and cracks propagated through the stir zone, revealing more cohesive fracture behavior. Finally, in Figure 6d (PA6-06), the joint exhibited a dense morphology and cohesive failure through the plastically deformed matrix, which is consistent with its higher shear and flexural strength. These observations confirm the direct effect of dwell time on the fracture mechanism.

### 3.2. Full Factorial Design Analysis Results

The effects of welding parameters (rotational speed, dwell time, and pin diameter) on joining strength were analyzed using a full factorial experimental design, and the ANOVA results are presented in Table 4. These results demonstrate that the selected factors significantly influence the joining strength, with the overall model being statistically significant (*p*-value: 0.001, F-value: 15.46). Among the parameters, rotational speed (rpm) has the most substantial effect, as evidenced by its *p*-value of 0.000, F-value of 27.78, and adjusted sum of squares (Adj SS) of 2227.73, accounting for the largest portion of the total variation. Dwell time (seconds) also shows a statistically significant impact on joining strength (*p*-value: 0.041, F-value: 6.22, Adj SS: 249.34), although its contribution is less pronounced compared to rotational speed. In contrast, pin diameter (Ø mm) does not have a statistically significant effect, as indicated by its high *p*-value (0.780) and low F-value (0.08). The error term’s adjusted mean square (Adj MS) of 40.10 indicates that the unexplained variability is within acceptable limits. Overall, rotational speed is identified as the most critical parameter, followed by dwell time, while pin diameter does not significantly affect the joining strength in this study. Although switching from M10 to M12 increases the local stir volume and can promote crystallinity, the shoulder-dominated heat input and dwell-controlled consolidation govern defect closure and interfacial bonding; therefore, pin diameter contributes little to the macroscopic strength compared with rotational speed and dwell time (Table 4).

**Figure 7:**
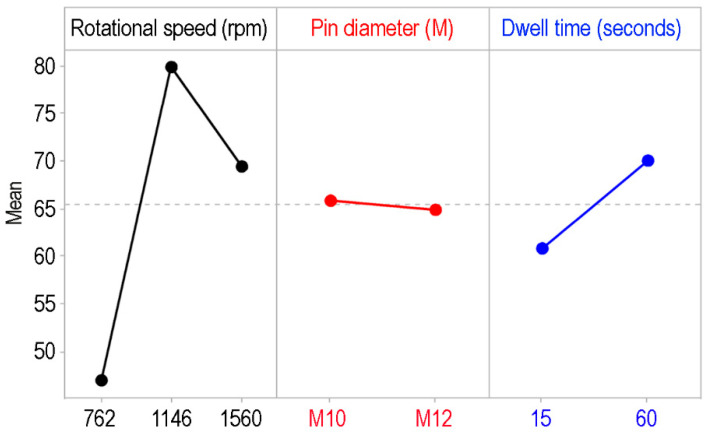
Main effects plot for tensile strength (MPa) of FSSW-joined PA6 samples.

Figure 7 presents the main effects plots. The main effects plot for tensile strength (MPa) illustrates the influence of rotational speed, pin diameter, and dwell time on the joining performance. Among these parameters, rotational speed exerts the most significant impact, with tensile strength increasing sharply from 762 rpm to 1146 rpm, where the maximum strength is achieved. However, at 1560 rpm, the strength decreases, indicating that 1146 rpm is the optimal speed for achieving higher tensile strength. Dwell time also exhibits a notable effect, with tensile strength improving substantially as the time increases from 15 s to 60 s, suggesting that longer dwell times enhance material bonding quality [11,12,13,14,15]. In contrast, pin diameter (M10 and M12) has minimal influence on tensile strength, as there is little variation between the two diameters. These findings align with the ANOVA results, confirming that rotational speed and dwell time are the critical parameters for tensile strength, whereas pin diameter does not significantly affect joining performance.

**Figure 8:**
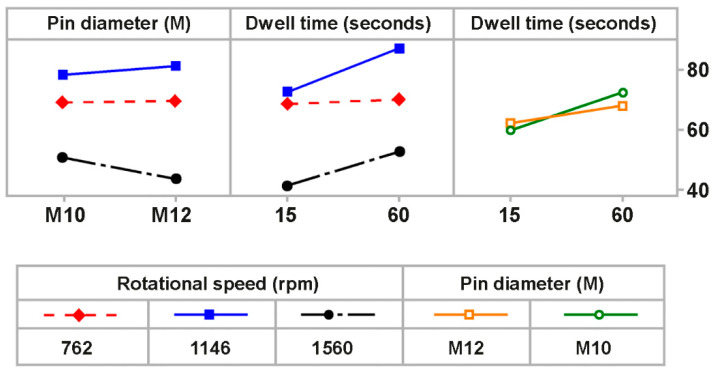
Interaction plot for tensile strength (MPa) of FSSW-joined PA6 samples.

Figure 8 presents the interaction plot for tensile strength (MPa), illustrating how rotational speed, pin diameter, and dwell time interact to influence joining performance. The plot shows that rotational speed has a significant effect on tensile strength, with 1146 rpm resulting in the highest tensile strength for both M10 and M12 pin diameters. However, tensile strength decreases at 1560 rpm. The effect of rotational speed is further influenced by dwell time, with a significant improvement in tensile strength observed at higher dwell times (60 s) when the optimal rotational speed of 1146 rpm is used. In contrast, minimal improvement is seen at 762 rpm and 1560 rpm. The interaction between pin diameter and dwell time shows only slight variation, with the M12 pin diameter slightly outperforming M10 at both dwell times. Overall, the plot confirms that rotational speed and dwell time are the most critical factors for tensile strength, while pin diameter has a minimal effect on joining performance, aligning with the findings from the ANOVA and the main effects plot.

**Figure 9:**
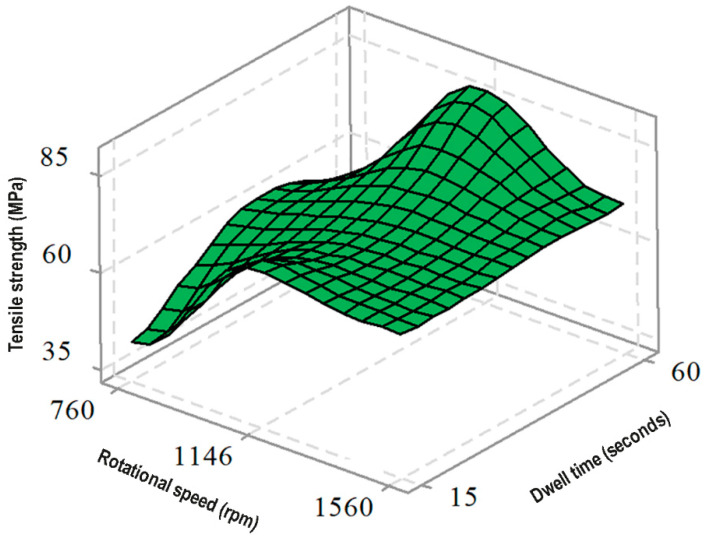
Surface plot of tensile strength as a function of rotational speed and mixing time.

Figure 9 illustrates the surface plot of tensile strength (MPa) as a function of rotational speed (rpm) and dwell time (seconds), providing a three-dimensional perspective on their combined effects on joining performance. The plot reveals that the maximum tensile strength is achieved at a rotational speed of 1146 rpm and a dwell time of 60 s, confirming these as the optimal parameters. At lower (760 rpm) or higher (1560 rpm) rotational speeds, tensile strength decreases, indicating the critical role of selecting an appropriate speed. Similarly, dwell time significantly affects tensile strength, with longer durations (60 s) resulting in improved bonding quality, whereas shorter durations (15 s) lead to lower tensile strength. The interplay between these parameters demonstrates that tensile strength can be maximized by simultaneously optimizing rotational speed and dwell time [11,15].

### 3.3. The Effect of Dwell Time on the Three-Point Bending Strength of Sheets Joined by FSSW

**Figure 10:**
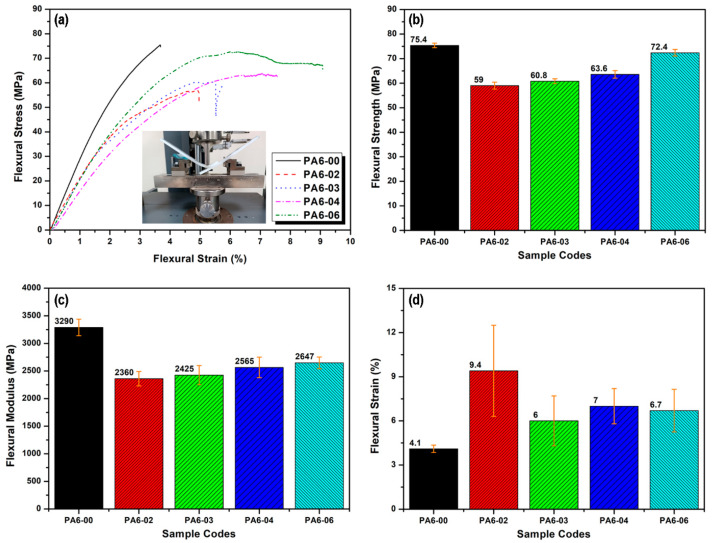
Effect of dwell time on the flexural properties of PA6 sheets joined by FSSW: (**a**) flexural curves, (**b**) strength, (**c**) modulus, and (**d**) strain.

The effect of dwell time on the flexural properties (flexural strength, modulus, and strain) of welded joints is illustrated in Figure 10. Increasing the dwell time significantly affected the flexural properties, including strength, modulus, and deformation behavior. The PA6-00 reference sample (not subjected to FSSW) exhibited the highest flexural strength (75.4 MPa) and modulus (3290 MPa), indicating that pure PA6 possesses high strength but limited deformation capacity. However, the mechanical performance of the PA6 samples subjected to FSSW changed notably with increasing dwell time.

For the PA6-02 sample with a 15 s dwell time, flexural strength decreased, but deformation capacity (strain) significantly increased. This suggests that the short dwell time hindered the formation of a homogeneous structure in the weld zone, leading to a more flexible structure that may also allow for greater energy absorption. For longer dwell times (PA6-03 and PA6-04), flexural strength increased to 60.8 MPa and 63.6 MPa, respectively, and the deformation capacity showed more balanced behavior (6% and 7%, respectively). This indicates that the extended dwell time promoted better homogenization of the material structure, improving the mechanical properties. For the PA6-06 sample, which was welded with the longest dwell time (60 s), flexural strength and modulus reached 72.4 MPa and 2647 MPa, respectively. Meanwhile, the deformation capacity was measured as 6.7%, and this sample exhibited overall balanced mechanical performance. It was found that short dwell times and insufficient thermomechanical processing hindered the formation of a homogeneous structure in the weld zone, adversely affecting the mechanical properties. In contrast, increasing the dwell time resulted in a more balanced strength and deformation capacity in PA6 materials, leading to optimized welding performance [8,11,24].

### 3.4. DSC Test Results of PA6

**Figure 11:**
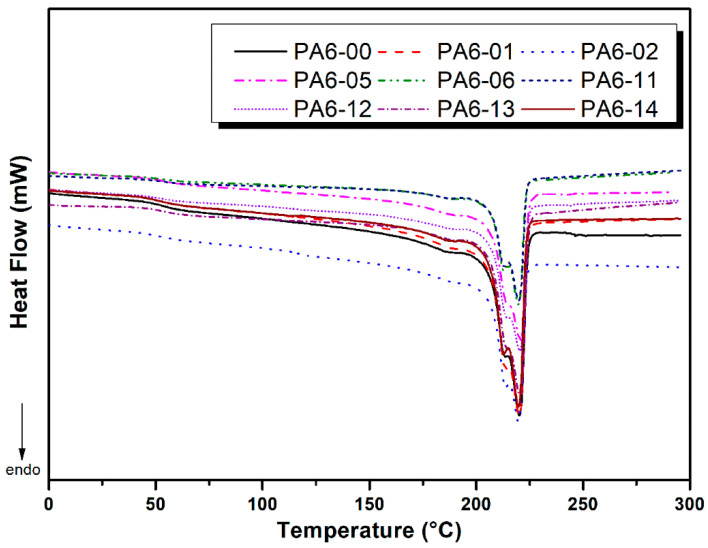
DSC curves of pure PA6 and FSSW-joined PA6 samples.

The DSC analysis results are presented in Table 5, with a graphical comparison shown in Figure 11. The results for PA6 samples joined using different FSSW parameters demonstrate that the applied welding conditions have notable effects on the material’s thermal properties. The reference sample, PA6-00, which was not subjected to any welding process, was included for comparison purposes and exhibited a moderate crystalline structure with a degree of crystallinity of 19.59%. The findings indicate that, in particular, dwell time and pin diameter markedly influence crystallization behavior [11,16,17,18]. For instance, PA6-06 (762 rpm, M12, 60 s) exhibited the highest degree of crystallinity (33.37%) and enthalpy value (76.76 J/g), whereas PA6-05 (762 rpm, M10, 60 s), processed under identical conditions except for a smaller pin diameter, showed only 15.73% crystallinity. This suggests that a larger pin diameter (M12) enhances material flow and thermal distribution, thereby promoting the formation of crystalline structures.

Moreover, while increasing the dwell time enhanced crystallinity in some samples, in others, this effect was limited. Interestingly, PA6-02 (762 rpm, M12, 15 s) demonstrated a high degree of crystallinity (31.50%) despite its short processing time, indicating that pin diameter and suitable rotational speed alone can significantly influence crystalline structure formation. Conversely, rotational speed alone did not appear to be a decisive factor for crystallinity but was found to affect the glass transition temperature (Tg). For example, PA6-11 (M10, 15 s), processed at 1560 rpm, exhibited the highest Tg value (68.31 °C), suggesting that higher rotational speeds restrict molecular mobility and thus increase Tg [35].

The λ (nm) values, representing crystalline lamella thickness, also varied across the samples. PA6-02 exhibited the thinnest lamellae at 13.86 nm, while PA6-01 had the thickest lamellae at 19.55 nm. Lower λ values typically correspond to more tightly packed and denser crystalline regions [36]. In this context, the combination of the lowest λ value and high degree of crystallinity (31.50%) observed in PA6-02 indicates the formation of thin yet dense crystalline lamellae. In contrast, PA6-01, with a higher λ value and relatively lower crystallinity (24.75%), likely developed thicker but less densely packed crystalline regions. These microstructural differences are consistent with the mechanical results: samples with thin and dense lamellae and higher crystallinity (e.g., PA6-02) exhibited higher stiffness but very low elongation at break, while samples with thicker lamellae and lower crystallinity (e.g., PA6-01) showed reduced strength but slightly improved ductility.

Overall, the FSSW parameters were found to notably affect the crystallization behavior and thermal characteristics of PA6. Specifically, dwell time, pin diameter, and rotational speed exert both individual and combined effects on the resulting structure. Optimizing these parameters may enable the tailoring of desirable thermal and structural properties in polymeric materials. It is noteworthy that the crystallinity increase observed with the larger pin diameter (M12) did not translate into higher tensile or shear strength. In this joint geometry, strength is controlled primarily by interfacial integrity and void minimization, processes driven more by rotational speed and dwell time than by pin diameter. This reconciles the limited statistical effect of pin diameter in ANOVA with its detectable influence on crystallinity.

### 3.5. Microstructure Analysis

**Figure 12:**
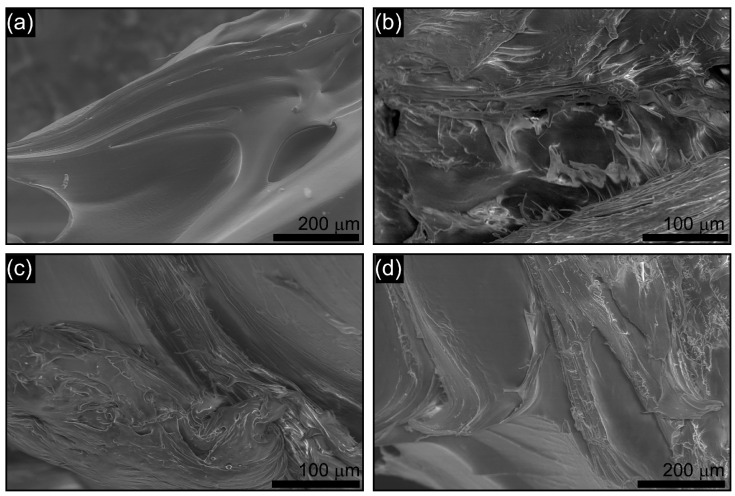
SEM micrographs of FSSW-welded PA6 samples: (**a**) PA6-01 (762 rpm, M10, 15 s), (**b**) PA6-02 (762 rpm, M12, 15 s), (**c**) PA6-04 (762 rpm, M12, 45 s), and (**d**) PA6-06 (762 rpm, M12, 60 s).

The SEM micrographs of selected welded samples (PA6-01, PA6-02, PA6-04, and PA6-06) are shown in Figure 12. Distinct morphological differences were observed depending on dwell time and pin diameter applied during the FSSW process. For PA6-01 (762 rpm, M10, 15 s), the weld region revealed insufficient material flow and weak interfacial bonding. The presence of voids and irregular flow lines indicates that the short dwell time and smaller pin diameter were not adequate to promote homogeneous mixing. This microstructural inhomogeneity is consistent with the low shear and fracture strength measured for this sample.

In PA6-02 (762 rpm, M12, 15 s), the microstructure appeared denser with fewer voids. The larger pin diameter enhanced material flow, resulting in better bonding even under short dwell conditions. However, localized defects were still visible along the stir zone boundary, correlating with its relatively high crystallinity (31.50%) but limited mechanical strength. The PA6-04 sample (762 rpm, M12, 45 s) exhibited a more compact and homogeneous morphology compared to PA6-01 and PA6-02. The extended dwell time improved polymer plasticization and mixing, reducing porosity and strengthening interfacial adhesion. This microstructural improvement is in good agreement with the enhanced shear and flexural performance of this sample. Finally, PA6-06 (762 rpm, M12, 60 s) displayed the most uniform morphology among the analyzed samples. The stir zone was dense and continuous, with minimal void formation. The combination of longer dwell time and larger pin diameter ensured sufficient thermal input and mechanical stirring, enabling effective molecular diffusion across the interface. This dense and defect-free morphology explains the highest crystallinity (33.37%) and superior mechanical performance obtained for PA6-06. Nevertheless, some localized traces of thermal–mechanical degradation were also visible, suggesting that excessively long dwell times may initiate polymer chain scission.

Overall, the SEM results confirm the findings of mechanical and DSC analyses: short dwell times produce voids and weak interfaces, whereas longer dwell times combined with larger pin diameters promote homogeneity, higher crystallinity, and improved bonding quality. These correlations demonstrate that dwell time and pin diameter are decisive parameters for weld integrity in the FSSW of PA6.

### 3.6. Thermal Monitoring of the FSSW Process

**Figure 13:**
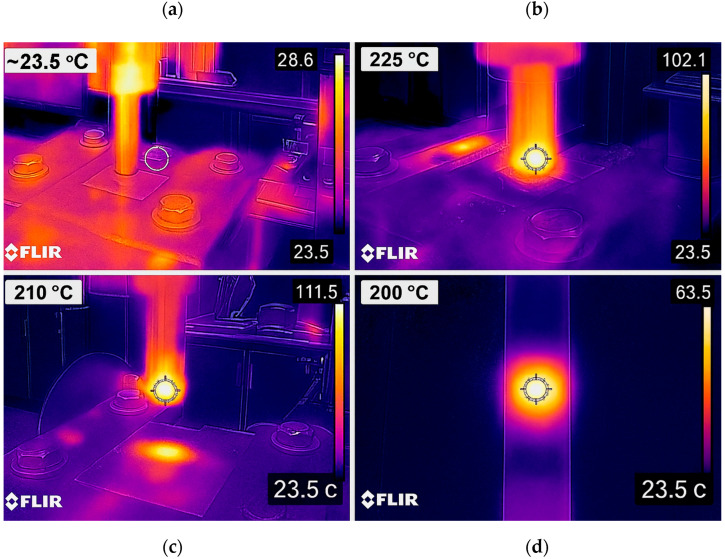
Thermal monitoring of PA6 sheets during FSSW: (**a**) initial stage, (**b**) plunging, (**c**) dwelling, and (**d**) cooling.

Thermal monitoring was carried out during the FSSW process using a FLIR E8 infrared camera to track the heat distribution and peak temperatures in the weld zone. Representative thermal images of the welding stages are shown in Figure 13.

At the initial stage, the PA6 sheets were at ambient temperature (~25 °C), without noticeable heat concentration. During the plunging stage, the shoulder–surface interaction generated rapid frictional heating, and the temperature at the tool–workpiece interface increased sharply to approximately 200–230 °C, which is near the melting temperature of PA6 but still within the safe softening range for solid-state welding. The heat flux was concentrated beneath the tool shoulder and radially dissipated outward, demonstrating localized plasticization of the polymer. In the dwell stage, the heat distribution became more uniform across the weld zone as continuous stirring promoted material mixing. The weld interface maintained a steady thermal field, preventing overheating and thus ensuring effective consolidation. The monitored temperatures confirmed that the thermal input remained below the degradation threshold of PA6 while being sufficient for joint formation. Finally, in the cooling stage, tool retraction was followed by a rapid temperature drop to below 50 °C within seconds, with residual heat concentrated in the weld center. This rapid cooling is critical, as it influences crystallization kinetics, lamella thickness, and ultimately the mechanical performance of the weld.

Overall, the thermal images illustrate that the FSSW process achieves a controlled heat flow localized to the weld zone, with sufficient softening for plastic deformation but without extensive melting. These findings are consistent with the characterization results, where dwell time and rotational speed governed weld integrity more strongly than pin diameter.

## 4. Conclusions

This study has demonstrated that friction stir spot welding (FSSW) significantly affects the mechanical, thermal, and microstructural properties of PA6, a widely used engineering plastic. Through a comprehensive analysis of various welding parameters, including rotational speed, dwell time, and pin diameter, key factors influencing the joining strength and structural integrity of PA6 were identified. Rotational speed emerged as the most influential parameter, with an optimal speed of 1146 rpm providing the highest tensile strength, while dwell time also proved critical for enhancing material bonding quality. The results of the interaction and surface plots further confirmed that combining appropriate rotational speed with sufficient dwell time maximizes tensile strength, providing insight into the optimal conditions for FSSW in PA6.

Additionally, the study revealed that the effect of welding parameters extends to the flexural properties and crystallization behavior of the material. Increasing dwell time led to improvements in flexural strength and deformation capacity, indicating the importance of achieving a balanced thermomechanical processing during welding. Furthermore, the crystallization behavior of PA6 was significantly influenced by pin diameter and mixing time, with larger pin diameters promoting higher crystallinity and better thermal distribution. The degree of crystallinity and crystalline lamella thickness (λ) varied across samples, indicating the potential to tailor the material’s thermal and structural properties by optimizing FSSW parameters.

In addition, thermal monitoring using infrared imaging confirmed that the generated heat remained localized to the weld zone and below the degradation threshold of PA6, ensuring controlled plasticization without excessive melting. This controlled heat input was shown to influence crystallization kinetics and mechanical performance, linking the thermal field directly with weld quality.

Moreover, SEM observations provided further microstructural evidence that short dwell times and small pin diameters led to voids and weak interfaces, while longer dwell times with larger pin diameters produced dense and homogeneous morphologies. These morphological differences correlated directly with the mechanical results, demonstrating the importance of combined thermal and microstructural assessments.

In conclusion, this research highlights the crucial role of process parameters in determining the mechanical and thermal performance of PA6 welded joints. By optimizing welding parameters, particularly rotational speed and dwell time, it is possible to enhance the material’s strength, flexibility, and thermal properties, making it more suitable for high-performance applications in industries such as automotive, aerospace, and electronics. The integration of mechanical testing, DSC, SEM, and thermal imaging confirms that a multi-perspective approach is essential for accurately understanding and optimizing FSSW in PA6. Future research should focus on further exploring the relationship between FSSW parameters and PA6’s microstructural development to refine welding techniques and broaden the material’s industrial applications.

## Figures and Tables

**Table 1:** FSSW joining parameters for PA6.

Sample Codes	Rotational Speed (rpm)	Pin Diameter (Ø mm)	Dwell Time (s)
PA6-00 (Reference)	-	-	-
PA6-01	762	M10	15
PA6-02	762	M12	15
PA6-03	762	M12	30
PA6-04	762	M12	45
PA6-05	762	M10	60
PA6-06	762	M12	60
PA6-07	1146	M10	15
PA6-08	1146	M12	15
PA6-09	1146	M10	60
PA6-10	1146	M12	60
PA6-11	1560	M10	15
PA6-12	1560	M12	15
PA6-13	1560	M10	60
PA6-14	1560	M12	60

**Table 2:** FFD factors and levels.

Factor	Unit	Level 1	Level 2	Level 3
Rotational speed	rpm	762	1146	1560
Dwell time	s	15	60	-
Pin diameter	M	10	12	-

**Table 3:** The lab shear test results.

Sample Codes	Elastic Modulus (MPa)	Tensile Strength (MPa)	Breaking Strength (MPa)	Elongation at Break (%)
PA6-00 (Reference)	3200 (±264)	73.4 (±1.0)	70.4 (±1.0)	476 (±63.4)
PA6-01	5410 (±342)	40.6 (±2.0)	8.8 (±1.4)	6.7 (±0.9)
PA6-02	9110 (±509)	41.3 (±3.0)	8.2 (±0.6)	6.8 (±0.9)
PA6-03	6310 (±174)	51.8 (±0.5)	10.3 (±0.1)	5.8 (±0.8)
PA6-04	9560 (±315)	56.5 (±2.0)	11.2 (±0.4)	4.8 (±0.6)
PA6-05	4960 (±420)	61.1 (±0.1)	12.2 (±0.2)	6.8 (±0.9)
PA6-06	5120 (±312)	64.2 (±1.0)	12.8 (±0.2)	6.8 (±0.9)
PA6-07	6983 (±304)	71.6 (±1.1)	14.3 (±0.3)	4.8 (±0.6)
PA6-08	7710 (±230)	73.3 (±0.6)	14.6 (±0.1)	4.8 (±0.6)
PA6-09	7160 (±431)	85.2 (±1.6)	18.3 (±3.3)	3.9 (±0.5)
PA6-10	7873 (±337)	89.2 (±1.1)	17.6 (±0.8)	3.9 (±0.5)
PA6-11	9980 (±627)	66.1 (±0.7)	13.1 (±0.2)	3.9 (±0.5)
PA6-12	9520 (±223)	68.7 (±1.3)	13.7 (±0.3)	3.9 (±0.5)
PA6-13	10,400 (±465)	69.3 (±0.8)	13.8 (±0.2)	5.8 (±0.8)
PA6-14	6840 (±530)	71.0 (±0.7)	14.2 (±0.2)	9.6 (±1.3)

**Table 4:** ANOVA for the full factorial design.

	DF	Seq SS	Contribution	Adj SS	Adj Ms	F-Value	*p*-Value
Rotational speed (rpm)	2	2227.73	80.68%	2227.73	1113.86	27.78	0.000
Pin diameter (M)	1	3.37	0.12%	3.37	3.37	0.08	0.780
Dwell time (seconds)	1	249.34	9.03%	249.34	249.34	6.22	0.041
Error	7	280.69	10.17%	280.69	40.10		
Total	11	2761.12	100.00%				

**Table 5:** The numerical data obtained from the DSC test.

Sample Code	ΔH (J/g)	Tg (°C)	Tm (°C)	Xc (%)	λ (nm)
PA6-00 (Reference)	45.05	56.66	221.57	19.59	16.08
PA6-01	56.93	56.25	223.07	24.75	19.55
PA6-02	72.46	33.71	220.22	31.50	13.86
PA6-05	36.19	51.28	222.44	15.73	17.93
PA6-06	76.76	50.25	220.96	33.37	14.99
PA6-11	51.92	68.31	221.13	22.57	15.28
PA6-12	63.63	41.89	222.26	27.67	17.51
PA6-13	55.52	49.04	222.59	24.14	18.29
PA6-14	39.12	44.45	222.24	17.01	17.46

## Data Availability

The original contributions presented in this study are included in the article. Further inquiries can be directed to the corresponding authors.
